# Genome Sequences of a Novel HIV-1 Circulating Recombinant Form (CRF59_01B) Identified among Men Who Have Sex with Men in Northeastern China

**DOI:** 10.1128/genomeA.00315-13

**Published:** 2013-06-06

**Authors:** Xiaoxu Han, Minghui An, Weiqing Zhang, Bin Zhao, Zhenxing Chu, Yutaka Takebe, Hong Shang

**Affiliations:** Key Laboratory of AIDS Immunology, Ministry of Health, Department of Laboratory Medicine, First Hospital of China Medical University, Shenyang, Chinaa; AIDS Research Center, National Institute of Infectious Diseases, Toyama, Shinjuku-ku, Tokyo, Japanb

## Abstract

We report here a novel HIV-1 circulating recombinant form (CRF) (CRF59_01B) comprised of CRF01_AE and subtype B, with two recombination breakpoints in the *pol* and *vpu-env* regions, respectively. CRF59_01B was identified from three epidemiologically unlinked men who have sex with men (MSM) in northeast China. This represents the second CRF identified in the MSM population in China.

## GENOME ANNOUNCEMENT

Human immunodeficiency virus type 1 (HIV-1) is classified into four groups: M, O, N, and P (1, 2). The group M strains, responsible for the vast majority of HIV infections in the world, comprise eleven subtypes and sub-subtypes, as well as 58 circulating recombinant forms (CRFs) and various types of unique recombinant forms (URFs) (see http://www.hiv.lanl.gov).

In Asia, 12 CRFs have been reported to date: CRF01_AE (3), CRF15_01B (4), and CRF34_01B (5) in Thailand, CRF07_BC (6), CRF08_BC (7), and CRF55_01B in China (8), CRF33_01B (9), CRF48_01B (10), CRF53_01B (11), and CRF54_01B (12) in Malaysia, CRF51_01B in Singapore (13), and CRF52_01B in Thailand and Malaysia (14). CRF51_01B and CRF55_01B are the CRFs identified among men who have sex with men (MSM).

Wide cocirculation and dual infection of CRF01_AE and subtype B in various geographical regions in Asia led to the emergence of various novel CRFs. We describe the genome sequences of a novel CRF (CRF59_01B) isolated from three epidemiologically unlinked MSM in northeastern China.

Near-full-length genome (NFLG) sequences (9.0 kb) were determined from plasma RNA using a single genome amplification method with two sets of primers designed for the determination of the 5′ and 3′ halves of the HIV-1 genome (15, 16). Amplicons were directly sequenced using the internal walking primers with an ABI 3730XL Sanger-based genetic analyzer. The study was approved by the Institutional Review Board of the First Affiliated Hospital of China Medical University.

The three NFLG sequences of CRF59_01B (accession no. KC462190, KC462191, and JX960635) were 8,804, 8,795, and 8,380 bp in size for strains 11CN.LNSY300392, 10CN.LNSY300533, and 09LNA423, respectively, spanning the noncoding region, the *gag, pol, env, tat, rev, vif, vpr, vpu*, and *nef* genes, and part of 3′ long terminal repeat (LTR). These three strains formed a distinct monophyletic cluster and did not belong to any known HIV-1 subtype or CRF. Bootscanning and informative site analyses (17) identified four unique recombination breakpoints between CRF01_AE and subtype B at the nucleotide positions (relative to HXB2) 2570 and 2719 in the *pol* region and 6149 and 8244 in the *vpu-env* region. These recombination breakpoints were shared among all three strains. Subregion tree analyses further confirmed the parental origin of each region of the recombinant genome as follows: region I (positions relative to HXB2: 790 to 2569) is CRF01_AE, region II (positions relative to HXB2: 2570 to 2718) is B, region III (positions relative to HXB2: 2719 to 6419) is CRF01_AE, region IV (positions relative to HXB2: 6149 to 8243) is B, and region V (positions relative to HXB2: 8244 to 9600) is CRF01_AE. The recombinant structure of CRF59_01B is indeed distinct from any previously reported CRFs. Subregion tree analyses also indicated that subtype B regions were of U.S. or European origin, unlike the subtype B′ (Thailand variant of subtype B) (3, 18) lineage associated with bloodborne epidemics in Asia (19), while CRF01_AE regions were of Thailand CRF01_AE origin, not related to the CRF01_AE variants (clusters 1 and 2) that we recently identified among MSM in China (15).

CRF59_01B is the second CRF identified in the MSM population in China. CRF55_01B was identified recently among MSM in southern China, while the new CRF described here was identified among MSM in northeastern China. The emergence of CRF55_01B and CRF59_01B suggests the extensive ongoing generation of new recombinant forms involving the CRF01_AE and subtype B lineages among MSM in various regions in China.

### Nucleotide sequence accession numbers. 

The sequences are available in GenBank under accession no. KC462190, KC462191, and JX960635.
